# Probiotic Microorganisms Inhibit Epithelial Cell Internalization of Botulinum Neurotoxin Serotype A

**DOI:** 10.3390/toxins8120377

**Published:** 2016-12-16

**Authors:** Tina I. Lam, Christina C. Tam, Larry H. Stanker, Luisa W. Cheng

**Affiliations:** Foodborne Toxin Detection and Prevention Research Unit, Western Regional Research Center, Agricultural Research Service, United States Department of Agriculture, 800 Buchanan Street, Albany, CA 94710, USA; tinaiunsanlam@gmail.com (T.I.L.); christina.tam@ars.usda.gov (C.C.T.); larry.stanker@ars.usda.gov (L.H.S.)

**Keywords:** botulinum toxin, foodborne toxins, probiotic bacteria, toxin absorption

## Abstract

Botulinum neurotoxins (BoNTs) are some of the most poisonous natural toxins known to man and are threats to public health and safety. Previous work from our laboratory showed that both BoNT serotype A complex and holotoxin can bind and transit through the intestinal epithelia to disseminate in the blood. The timing of BoNT/A toxin internalization was shown to be comparable in both the Caco-2 in vitro cell culture and in the oral mouse intoxication models. Probiotic microorganisms have been extensively studied for their beneficial effects in not only maintaining the normal gut mucosa but also protection from allergens, pathogens, and toxins. In this study, we evaluate whether probiotic microorganisms will block BoNT/A uptake in the in vitro cell culture system using Caco-2 cells. Several probiotics tested (*Saccharomyces boulardii*, *Lactobacillus acidophilus*, *Lactobacillus rhamnosus* LGG, and *Lactobacillus reuteri*) blocked BoNT/A uptake in a dose-dependent manner whereas a non-probiotic strain of *Escherichia coli* did not. We also showed that inhibition of BoNT/A uptake was not due to the degradation of BoNT/A nor by sequestration of toxin via binding to probiotics. These results show for the first time that probiotic treatment can inhibit BoNT/A binding and internalization in vitro and may lead to the development of new therapies.

## 1. Introduction

Botulinum neurotoxins are produced by the ubiquitous, gram-positive, anaerobic spore-forming *Clostridium* species and are the causative agent of botulism [1,2]. There are at least seven, possibly eight, different serotypes of BoNTs (A–H) of which A, B, E, and F are known causes of botulism in humans [3,4,5,6,7]. BoNTs are highly poisonous to humans with a parenteral lethal dosage of 0.1–1 ng/kg and an oral dose of 1 μg/kg. They are classified by the Centers for Disease Control and Prevention (CDC) as among the highest threats for bioterrorism (Tier 1 Category A agents). Additionally, BoNTs remain a public health and safety threat in the form of foodborne, wound, and infant botulism. Due to its mortality and morbidity, there is a significant economic burden associated with the long-term management of intoxication.

BoNTs are A-B dimeric toxins synthesized as ~150 kDa holotoxin with a heavy chain ~100 kDa linked by a disulfide bond to the light chain ~50 kDa. There are three functional domains: a receptor binding domain (H_C_), translocation domain (H_N_), and a catalytic domain (LC) [7]. The preferential target cells for BoNTs are the peripheral cholinergic neurons. Binding of H_C_ to carbohydrate and protein receptors on the presynaptic membrane results in BoNT endocytosis [8,9,10]. Internalization of BoNTs leads to H_N_ pore formation in the endosomal membrane resulting in the translocation of the catalytic domain LC into the cytosol [11,12,13,14]. The catalytic domain LC is a zinc-dependent endopeptidase that cleaves proteins associated with intracellular vesicular transport such as SNAP-25 (synaptosome-associated protein of 25 kDa), VAMP (vesicle-associated membrane protein), or syntaxin [15,16,17]. Due to the cleavage of these mediators of intracellular transport, exocytosis of the neurotransmitter acetylcholine from neurons is inhibited causing flaccid muscle paralysis. In foodborne illnesses caused by BoNTs, toxins must be able to survive initially in the lumen of the gastrointestinal tract, then bind and translocate through the intestinal epithelium to reach the bloodstream. Previous work from our laboratory showed that the BoNT/A complex, comprised of the combination of holotoxin with neurotoxin-associated proteins (NAPs), binds and transits through the intestinal epithelia to disseminate in the blood faster than BoNT/A holotoxin alone [18]. Therefore, understanding the mechanism(s) in which BoNTs bind to and breach this epithelial barrier is of great scientific interest because of the potential development of new therapeutics to inhibit this required first step of oral intoxication.

The gastrointestinal tract (GI) has evolved as one of the largest barriers to segregate the extracellular milieu from mammalian cells. Colonization of the gastrointestinal tract by a variety of commensal bacteria aid in not only the digestion and absorption of nutrients but also the development and regulation of the mucosal immune system [19]. There are anywhere between 10^10^ to 10^12^ colony-forming units per gram of intestinal content in the colon and 60% of all fecal matter mass in humans is due to bacteria [20]. The colonization of the GI tract with microbes carries with it the risk of infection and inflammation if the barrier between the microorganisms and hosts is damaged. The intestinal epithelium acts as a physical and biochemical barrier to not only commensal and pathogenic bacteria but also to all other luminal contents including other injurious matter such as toxins. Specialized intestinal epithelial cells (IECs) are able to sense and respond to these stimuli with appropriate responses such as increasing their barrier function to activation of anti-pathogenic immune mechanisms [19].

Probiotics, as defined by the World Health Organization (WHO), are live microorganisms that provide health benefits to hosts when ingested in adequate amounts. They have been shown to have potential significant therapeutic value for a range of diseases such as *H. pylori* infection, irritable bowel syndrome, and inflammatory bowel disease (ulcerative colitis and Crohn’s disease) as well as boosting the immune system of healthy individuals [21,22,23,24,25,26]. The most common probiotic strains used are *Lactobacillus*, *Bifidobacteria*, and the yeast strain *Saccharomyces cerevisiae* var *boulardii* (SB). Lactic acid bacteria and bifidobacteria have been shown to remove heavy metals [27], cyanotoxins [28], and mycotoxin from in vitro aqueous solutions [29,30]. The probiotic effects seen are both strain and species dependent indicating that combinations of different strains and species may need to be tailored to the specific issue at hand rather than having one “universal” probiotic therapy. Though some beneficial effects of probiotics have been shown in both in vivo and in vitro studies, the exact mechanism(s) that is responsible for these beneficial effects remains to be fully elucidated. The mechanisms that have been attributed to probiotics are: (a) maintenance of the gut epithelial barrier, (b) competitive exclusion of pathogenic organisms, (c) secretion of antimicrobial products, and (d) regulation of the mucosal immune system in favor of the hosts.

Since probiotics have been shown to block pathogen internalization as well as remove heavy metals and some toxins, we wondered if probiotics may block entry and subsequent internalization of BoNT/A in an in vitro cell based assay system using Caco-2 cells.

## 2. Results

### 2.1. The Effect of Pre-Treatment with Saccharomyces Boulardii on BoNT/A Uptake in Caco-2 Cells

Previous work in our laboratory established two in vitro Caco-2 cell models to test the entry and subsequent internalization of BoNT/A holotoxin and BoNT/A complex (AC) [18]. This study showed that BoNT/A entry and internalization was enhanced by the presence of neurotoxin-associated proteins in the BoNT/A complex. Significant internalization of toxin complex was achieved by 4 h post-intoxication whereas holotoxin was slightly delayed. Since some probiotics have been shown to be important for inhibition of pathogens as well as toxin binding to host mammalian cells, we wondered if pre-treatment with the probiotic yeast strain *Saccharomyces boulardii* (SB) would have a negative effect on BoNT/A binding and internalization in Caco-2 cells.

We chose a simple Caco-2 cell model to study the effect of probiotics on BoNT/A entry. This in vitro model was shown to mirror results found through the in vitro polarized Caco-2 epithelial cell and in the mouse oral intoxication model [18]. Caco-2 cells were either pre-treated with media (control) or SB low (10^4^ CFU) or high (10^8^ CFU) concentrations for 30 min at 37 °C before removal of non-adherent SB and subsequent washing with 1× HBSS three times. Cells were then incubated with 50 ng/mL of BoNT/A toxin complex at 37 °C for 4 h. At the end of this incubation, cells were washed and fixed with 4% paraformaldehyde. Immunostaining was performed to detect BoNT/A toxin using a polyclonal rabbit anti-BoNT/A antibody with detection using a goat anti-rabbit-IgG-Alexa-488. Additionally, these coverslips were stained with Rhodamine-Phalloidin to delineate the actin cytoskeleton in mammalian cells. DAPI was used to stain nuclear DNA. Images were obtained throughout the depth of the cells to measure the internalization of BoNT/A. Mean fluorescence intensities were measured throughout the depth of each field of cells (Z stack) containing the same area. Mean intensity multiplied by area for each Z section was calculated and the sum of the total fluorescence was defined as BoNT/A signal indicating cellular uptake. The mean of the BoNT/A signal was calculated and the statistical significance was determined for each condition.

**Figure 1:**
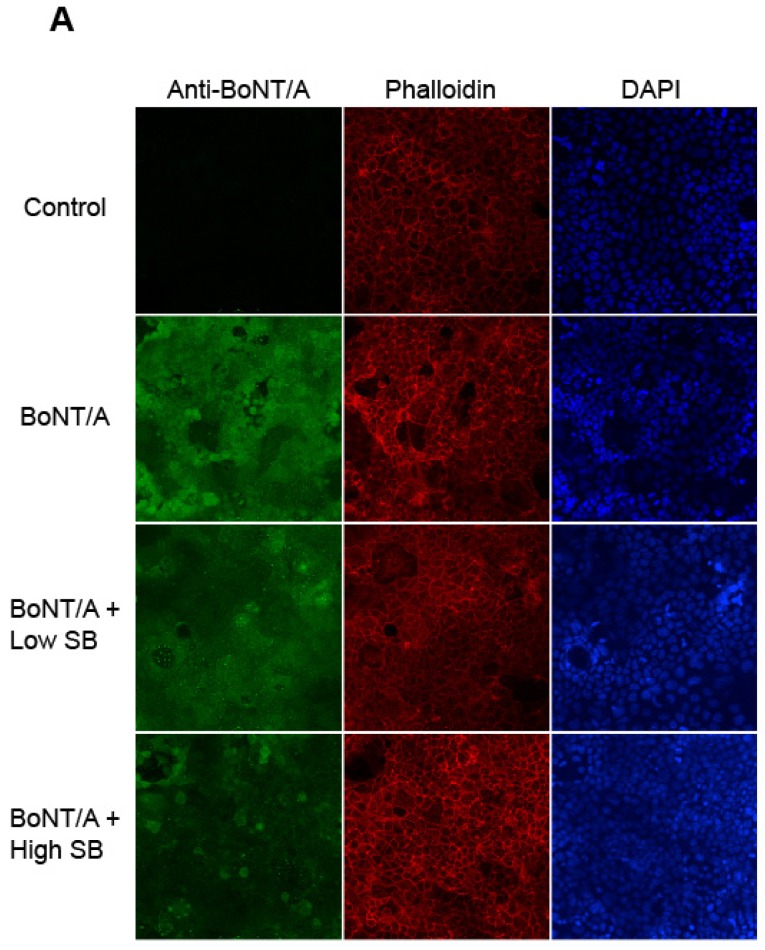

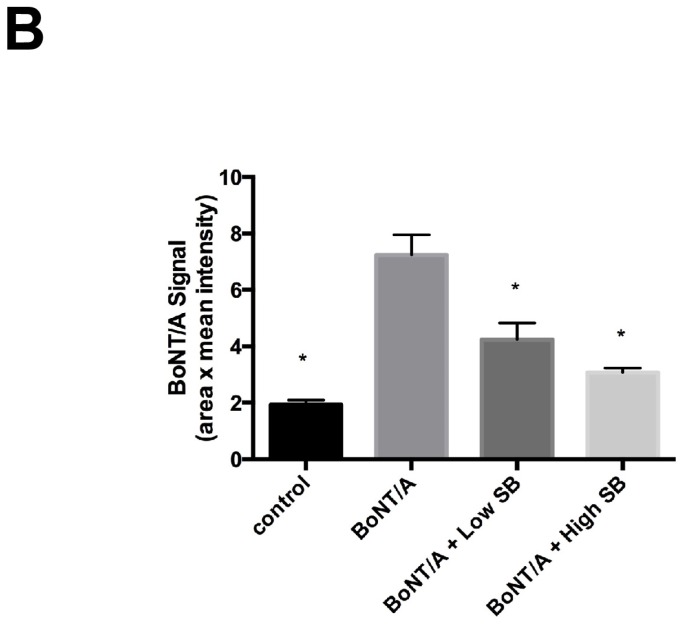
Internalization of BoNT/A into Caco-2 cells is significantly reduced in a dose-dependent manner by pre-treatment with the probiotic *Saccharomyces boulardii*. (**A**) Caco-2 cells were treated with media (control) or BoNT/A complex for 4 h at 37 °C. Some Caco-2 cells were either pre-treated with *Saccharomyces boulardii* (SB*)* for 30 min at 37 °C at either high SB (10^8^ CFU) or low SB (10^4^ CFU) before the addition of BoNT/A. Cells were fixed and stained with Alexa-488 labeled antibodies to BoNT/A, DAPI (nuclear), and Rhodamine-Phalloidin (actin cytoskeleton). Representative images at 40− magnification are shown; (**B**) The cellular uptake of BoNT/A was quantified by determining the mean fluorescence of three randomly chosen optical fields from each of four coverslips per experiment using ImageJ software. Values represent means of four independent experiments ± SEM. Statistical significance was determined using two-way ANOVA followed by the Tukey-Kramer test where multiple groups that are compared with *p*-values < 0.05 are taken to indicate significant differences between groups (*).

A statistically significant cellular uptake of BoNT/A after 4 h incubation of BoNT/A toxin complex as compared to the control treated with media alone (BoNT/A vs. control, *p* = 0.0004) is seen in Figure 1A. BoNT/A present in Caco-2 cells was visualized with bright green fluorescence while the actin cytoskeleton was stained in red and DAPI stained the cellular nuclei blue. Signal intensity for the different conditions were quantified in Figure 1B. Figure 1 shows that pre-treatment with SB 30 min prior to toxin addition has a negative effect on BoNT/A uptake. The addition of 10^8^ CFU of SB reduces the BoNT/A fluorescence signal (decrease in green fluorescence) even more than 10^4^ CFU, indicating that there is a dose-dependent effect on internalization and that it is statistically significant (BoNT/A vs. BoNT/A + Low SB, *p* = 0.0181; BoNT/A vs. BoNT/A + High SB, *p* = 0.0013).

### 2.2. The Effect of Pre-Treatment with Escherichia coli MG1655 on BoNT/A Uptake in Caco-2 Cells

**Figure 2:**
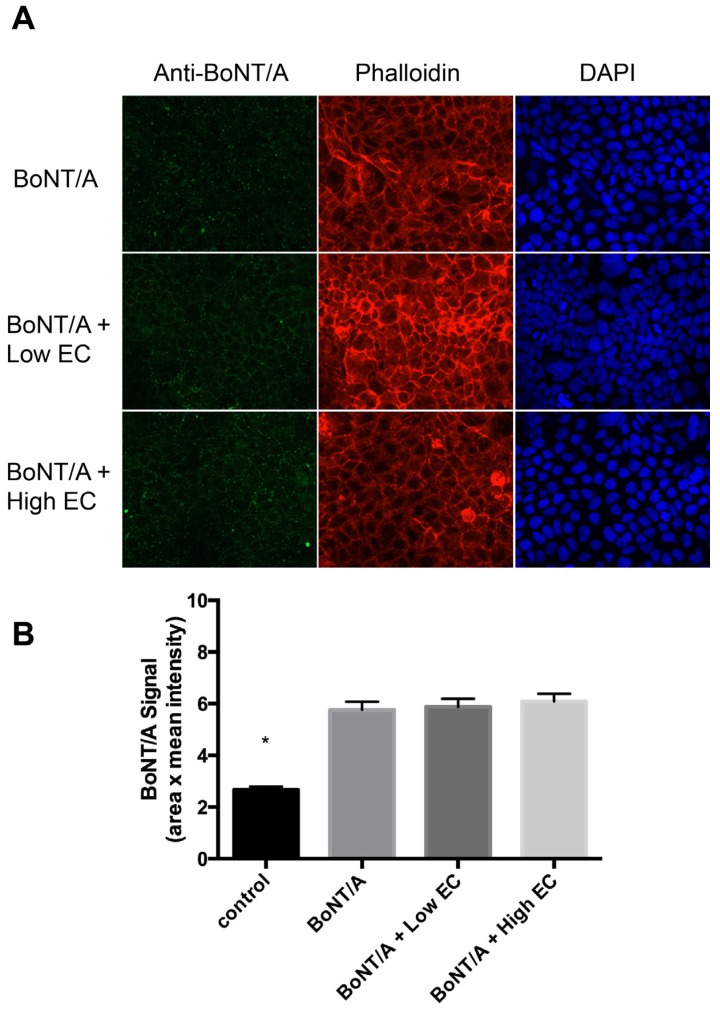
Pre-treatment with *Escherichia coli* MG1655 does not affect the internalization of BoNT/A into Caco-2. (**A**) Caco-2 cells were treated with media (control) or BoNT/A complex for 4 h at 37 °C. Some Caco-2 cells were either pre-treated with *Escherichia coli* (EC) for 30 min at 37 °C at either high EC (10^8^ CFU) or low EC (10^4^ CFU) before the addition of toxin. Cells were fixed and stained with Alexa-488 labeled antibodies to BoNT/A, DAPI (nuclear), and Rhodamine-Phalloidin (actin cytoskeleton). Representative images at 40− magnification are shown; (**B**) The cellular uptake of BoNT/A was quantified by determining the mean fluorescence of three randomly chosen optical fields from each of three coverslips per experiment acquired using a Zeiss Axio Observer.Z1 with Apotome.2 and analyzed with Zeiss Zen Pro 2012 software. Values represent means of four independent experiments ± SEM**.** Statistical significance was determined using two-way ANOVA followed by the Tukey-Kramer test where multiple groups that are compared with *p*-values < 0.05 are taken to indicate significant differences between groups (*). There is no statistical significance with pretreatment with EC.

Since we see a significant reduction on the internalization of BoNT/A into Caco-2 cells with SB in a dose-dependent manner, one can argue that this decrease in cellular uptake may be inherently due to non-specific interactions between any microorganism given in sufficient quantities. To assess whether this hypothesis was true, we asked whether a non-probiotic strain of *Escherichia coli* K12 MG1655 (EC) can reduce the uptake of BoNT/A in Caco-2 cells. As seen in Figure 2A,B, we show that EC does not have any statistically significant effect on BoNT/A uptake either at a low dose (10^4^ CFU) or a high dose (10^8^ CFU) unlike SB (Figure 1A,B). However, a statistical difference was seen between the control media alone compared to the sample treated with BoNT/A complex (control vs. BoNT/A, *p* < 0.0001). These results support the hypothesis that probiotics can have a beneficial effect on blocking the internalization of the foodborne toxin botulinum neurotoxin serotype A.

### 2.3. The Effect of Pre-Treatment with Lactobacillus Acidophilus, Lactobacillus rhamnosus LGG, and Lactobacillus Reuteri on BoNT/A Uptake in Caco-2 Cells

We showed in Figure 1 that the probiotic yeast strain SB has a beneficial effect on decreasing the uptake of BoNT/A in our in vitro cell culture system. We wanted to ask if other probiotic strains such as Lactobacilli would have a similar effect because some Lactobacilli strains such as *Lactobacillus acidophilus* (LA), *Lactobacillus rhamnosus* (LGG), and *Lactobacillus reuteri* (Lr) have been shown to have beneficial effects on host physiology against pathogen and toxin injuries.

**Figure 3:**
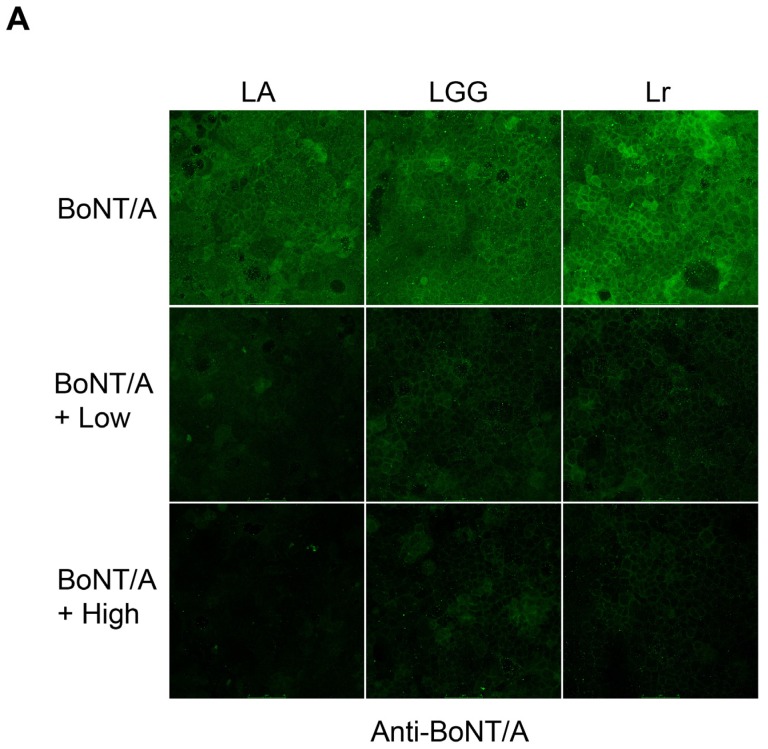

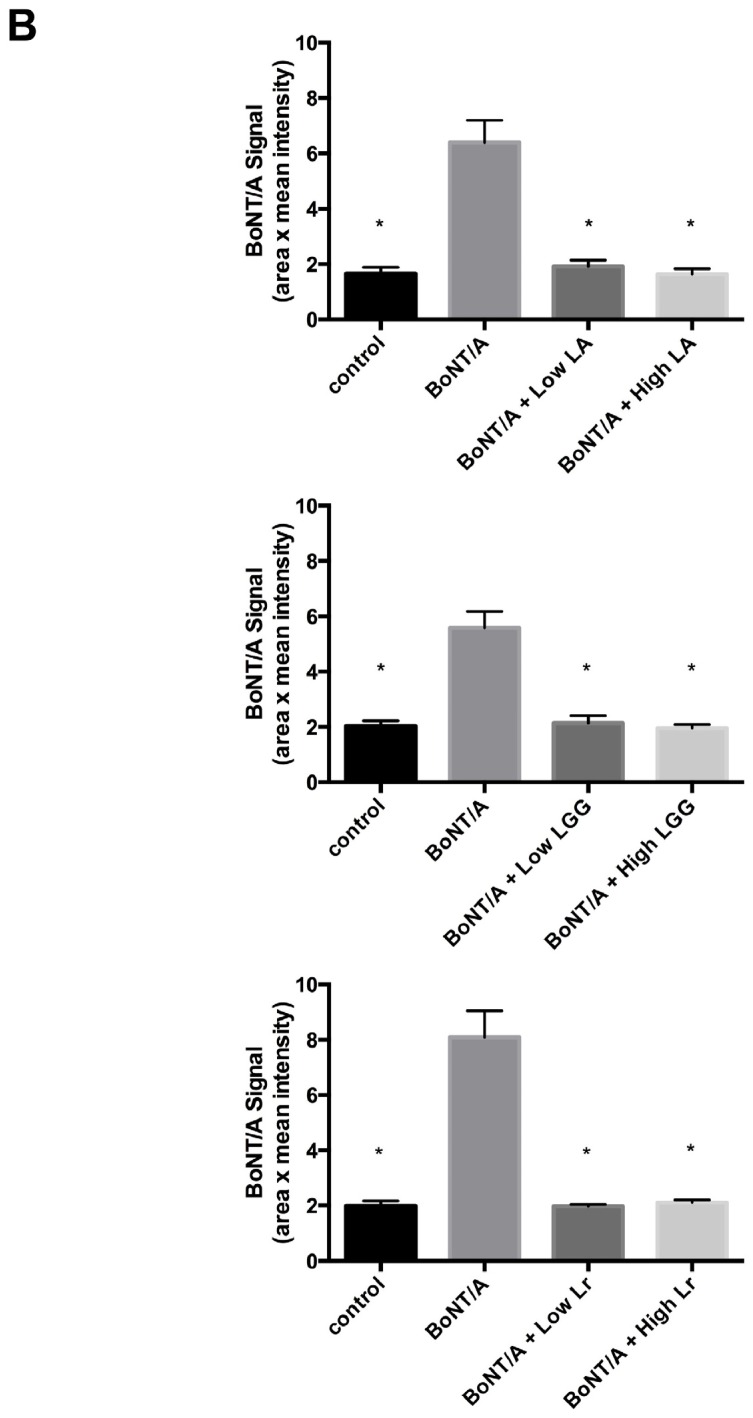
Pre-treatment with the probiotic *Lactobacillus acidophilus*, *Lactobacillus rhamnosus*, and *Lactobacillus reuteri* blocks internalization of BoNT/A into Caco-2 cells. (**A**) Caco-2 cells were treated with media (control) or with BoNT/A for 4 h at 37 °C. Some Caco-2 cells were either pre-treated with *Lactobacillus acidophilus* (LA)*, Lactobacillus rhamnosus* (LGG), *or Lactobacillus reuteri* (Lr) for 30 min at 37°C at either high (10^8^ CFU) or low (10^4^ CFU) before addition of BoNT/A complex. Cells were fixed and stained with Alexa-488 labeled antibodies to BoNT/A, DAPI (nuclear), and Rhodamine-Phalloidin (actin cytoskeleton). Representative images at 40− magnification showing BoNT/A fluorescence are shown; (**B**) The cellular uptake of BoNT/A was quantified by determining the mean fluorescence of three randomly chosen optical fields from each of the four coverslips per strain per experiment using ImageJ software. Values represent means of four independent experiments ± SEM**.** Statistical significance was determined using two-way ANOVA followed by the Tukey-Kramer test where multiple groups are compared with *p*-values < 0.05 are taken to indicate significant differences between groups (*).

Lactobacilli treatment severely reduces BoNT/A uptake into Caco-2 cells (Figure 3A,B). This severe inhibition of BoNT/A internalization by LA, LGG, and Lr is more dramatic than with SB (Figure 3A,B vs. Figure 1A,B). Additionally, the dose-dependent BoNT/A uptake decrease seen with SB does not seem to happen with LA, LGG, or Lr. A low dose (10^4^ CFU) of LA, LGG, and Lr is sufficient to almost completely block BoNT/A internalization to the levels seen in control cells which have not seen toxin (BoNT/A vs. LA Low, *p* = 0.0018; BoNT/A vs. LGG Low, *p* = 0.0019; BoNT/A vs. Lr Low, *p* = 0.0007). These results suggest that there may be differences in the ability of various probiotic strains to efficiently block some foodborne toxins such as BoNT/A.

### 2.4. Evaluation of the Mechanism Used by Probiotic Strains to Block BoNT/A Internalization

We have shown that treatment with SB, LA, LGG, and Lr strains prior to the addition of BoNT/A complex reduced the internalization of BoNT/A toxin in a colonic adenocarcinoma cell model. However, the mechanism(s) used by probiotics to block BoNT/A internalization is still unclear. One potential mechanism of action would be for these probiotics to secrete proteases that could degrade BoNT/A and hence there would be less BoNT/A to bind to and be internalized into the cells. Another mode of action would be for non-specific binding of BoNT/A to the probiotics themselves (i.e., cell walls) thus sequestering BoNT/A from its cellular receptors and not allowing for binding and subsequent internalization. A third potential mechanism is for the probiotics to compete for binding with BoNT/A to its cellular receptors, thus blocking binding and internalization.

We sought to answer this important question using a co-precipitation assay with the results detected using an antibody that recognizes BoNT/A in Western blots. BoNT/A (2 μg/mL) was added to an aliquot of washed overnight bacterial cultures (EC, SB, LA, LGG, and Lr) in 1× HBSS and then incubated for 4 h at 37 °C. The samples were centrifuged to fractionate BoNT/A into soluble supernatant (unbound) and insoluble pellet (bound). Protein samples from each fraction were TCA-precipitated, solubilized in protein sample buffer, and prepared for SDS-PAGE electrophoresis. Proteins were transferred onto PVDF membrane and immunoblotting was performed. A rabbit polyclonal anti-BoNT/A was used to bind to BoNT/A and detection was enabled by the addition of a goat anti-rabbit IgG conjugated to horseradish-peroxidase. Chemiluminescent substrate was added and signal was detected using an AlphaImager. Densitometry was used to detect signal intensity using the FluorChemSP.

**Figure 4:**
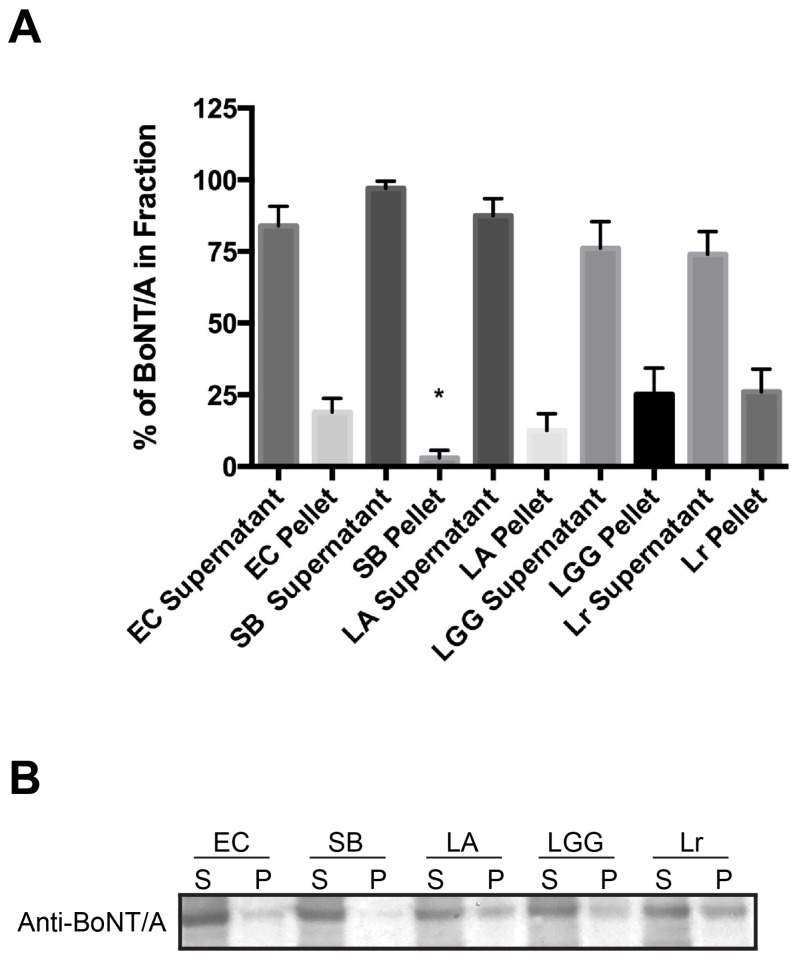
Decreased cellular uptake of BoNT/A complex is not due to the proteolytic degradation of holotoxin nor binding of toxin to probiotics. BoNT/A was added to either *Escherichia coli* MG1655, or probiotics and incubated for 4 h at 37 °C. Soluble supernatant (S) and insoluble pellet (P) fractions were precipitated with trichloracetic acid (TCA). Precipitates were solubilized with sample loading buffer and loaded onto 10% Bis-Tris NuPage gels. Gels were transferred onto PVDF membranes and incubated with primary polyclonal antibody to BoNT/A (Metabiologics) and secondary goat anti-rabbit-HRP. Western blot was developed using Pierce SuperSignal ECL substrate. (**A**) Mean percent signal of BoNT/A in each fraction was quantified from four independent experiments ± SEM using FluorChem SP (Alpha Innotech); (**B**) Representative Western depicting the presence of full length BoNT/A. Statistical significance was determined by a two-tailed unpaired Student’s *t*-test, (*) *p* < 0.05.

In Figure 4B, full length BoNT/A is predominately detected in Western blots at the expected size of ~150 kDa whether treated with EC, SB, LA, LGG, or Lr. This result suggests that the mechanism of action with any of the probiotics to decrease BoNT/A internalization is not due to the degradation of BoNT/A by secreted probiotic proteases (Figure 4A,B). As expected, treatment with EC, a non-probiotic strain that does not decrease BoNT/A uptake in cells, fractionates the BoNT/A mainly in the soluble supernatant unbound fraction ~83% with ~17% found in the insoluble bound pellet. BoNT/A is found exclusively in the soluble supernatant fraction and not bound to SB in the pellet (EC pellet vs. SB pellet, *p* = 0.0272). LA, LGG, and Lr were found to be even more efficient than SB in inhibiting BoNT/A uptake and all three show BoNT/A is present predominately in the soluble supernatant fraction similar to EC. These results suggest that probiotics themselves are not non-specifically sequestering toxins from mammalian cells. Thus, the most likely mechanism of action is due to competition between the probiotics and BoNT/A for the same cellular receptors either directly or via steric-hindrance.

## 3. Discussion

Botulinum neurotoxins, with their potential contamination of food, are bioterror threats as well as public health hazards. Consumption of botulinum neurotoxins from food sources leads to muscle paralysis and/or death for humans. There is a significant economic burden due to botulinum intoxication because of the need for long term supportive care and intensive hospitalization associated with this disease. Therefore, studies to elucidate the initial entry and internalization process of the toxin in the gut is of critical importance because of the potential development of new therapies to proactively block intoxication or in ameliorating the function of the toxin after ingestion.

BoNTs also cause infant botulism, which is usually associated with the ingestion of foods contaminated with *Clostridia* spores. Ingestion and subsequent germination of these spores into viable neurotoxigenic bacteria that are able to colonize the infant gastrointestinal system due to the lack of a robust gut microbiota to outcompete *Clostridia* [1,31]. BoNTs after ingestion or in situ production from bacteria must be able to survive in the lumen of the gastrointestinal tract and then traverse the intestinal epithelium from the apical to the basolateral side to reach its target cells.

The mechanism(s) as to how this occurs has been a major focus in the field. Previous work showed that the majority of toxin absorption occurs in the mouse upper small intestine [7,32]. One model for the transit of BoNTs suggests that the holotoxin itself can transcytose through the intestinal epithelium [33,34,35]. A second model for the BoNT absorption from the epithelium implicates the hemagglutin proteins (HA) in this process by binding cell surface receptors on the apical side, transcytosis, and potential disruption of the epithelial barrier at the basolateral side to allow for paracellular transport of the toxins in certain situations [33,36]. Our lab has shown in in vitro and in vivo intoxication models that neurotoxin-accessory proteins enhanced the rate of entry of BoNT/A in comparison to holotoxin alone, and entry was localized first to intestinal villi and subsequently to the intestinal crypts [18].

The major defensive mechanism of the gut is thus the intestinal barrier, which maintains epithelial integrity, and protects the host from the environment. In defense of this barrier, there are also the mucous layer, antimicrobial peptides, secretory IgA, and the epithelial junction adhesion complex [37]. Disruption of this barrier allows for bacteria and food antigens to reach the submucosa, which can induce an inflammatory response potentially leading to the intestinal disorders such as inflammatory bowel disease [38,39]. Probiotic treatment has been shown to have many beneficial effects including: (a) therapeutic treatment for human diseases, (b) inhibition of growth and toxin production for pathogens, and (c) extraction of heavy metals and toxins (aflatoxin B1) from solution.

Studies have suggested that probiotics enhance the expression of genes involved in tight junction signaling as a possible mechanism to reinforce the integrity of the intestinal epithelium [40]. An example of this is that Lactobacilli treatment in a T84 cell barrier model modulates several genes such as E-cadherin and β-catenin that affect adherence cell junctions. Lactobacilli treatment of intestinal cells also differentially regulates the phosphorylation of adherence junction proteins and the abundance of protein kinase C (PKC) isoforms, such as PKCδ, thereby positively reinforcing epithelial barrier function [41]. Not only is the epithelial barrier reinforced before damage, work with the probiotic *Escherichia coli* Nissle 1917 strain (EcN1917) suggests that it can initiate repair of the mucosal barrier after damage by enteropathogenic *E. coli* in T84 and Caco-2 cells by enhancing the expression and redistribution of tight junction proteins of the zonula occludens (ZO-2) and PKC [42,43]. Similar repair mechanisms have been reported with treatment with *Lactobacillus casei* DN-114001 [44] and VSL3 (a pre- and probiotics mixture) [45].

Studies have also shown that probiotics are able to modify their environment to make it more hostile to their potential competitors. The production of antimicrobial substances such as lactic and acetic acid is one example of this modification. *Lactobacillus* cocultivation with *E. coli* O157:H7 in broth culture produced organic acids which lead to a decrease in both pH and *stx_2A_* expression [46].

We have shown that pre-treatment with the yeast strain *Saccharomyces boulardii* significantly decreased BoNT/A binding and internalization in Caco-2 cells after 4 h in a dose-dependent and specific manner whereas the control non-probiotic strain *E. coli* did not (Figure 1A,B vs. Figure 2A,B). Treatment with *Lactobacillus acidophilus*, *Lactobacillus rhamnosus* LGG, and *Lactobacillus reuteri* demonstrated an even greater protective effect than *Saccharomyces boulardii* by almost completely abolishing BoNT/A binding and internalization at the lower 10^4^ CFU dose (Figure 3A,B vs. Figure 1A,B). We have also tested the inhibition of BoNT/A binding using different commercial probiotic supplements, most showed inhibitory effects (data not shown). These results suggest that, consistent with other probiotic studies, the beneficial effects of probiotics are strain- and species-dependent [47,48]. The probiotic *E. coli* strain Nissle 1917 was however not available in the U.S. for use in comparison testing at the time of study. Further research using probiotic *E. coli* or comparable strains are needed to elucidate the mechanism of inhibition. Future studies are also needed to determine the right formulation or combination of probiotic strains for optimal toxin entry inhibition and the specific mechanisms of toxin entry inhibition.

What role do probiotic organisms play in the defense of the intestinal epithelium against toxic invader and block BoNT/A entry? One hypothesis would be that the toxin itself could be degraded by the probiotics via secretion of proteases, thus rendering the toxin unable to bind its cellular receptors. An alternative theory would be that the probiotics would non-specifically bind BoNT/A itself due to some constituent of their cell walls, thus titrating the toxin from the host cells. A third potential mechanism would competitive inhibition between the probiotics and BoNT/A for binding to the host cell either through direct exclusion of BoNT/A from binding to the host cell receptors or indirect exclusion of BoNT/A due to steric hindrance from probiotic binding to the cell membrane. The adhesive properties due to the interactions between surface proteins and mucins may be utilized by some probiotic strains as an antagonistic mechanism against gastrointestinal pathogens as well as some toxins. Since binding to the mucous layer and intestinal cells is required for entry and colonization by many pathogens and toxins, mechanisms that will inhibit this first required step are critical for disease prevention. To prevent enteric infections, approaches such as (a) the development of synthetic oligosaccharide-based anti-infectives such as Synsorb (inert silica particles-linked to synthetic oligosaccharides) have been developed against the following: Stx1/2-Gb_3_, Stx2e-Gb_4_, Ctx-GM1, LT-GM1, epsilon toxin-GM2, TcdA-Lewis X and Lewis Y, botulinum neurotoxin- GD1a, GT1b, *E. coli* K88 ad fimbriae-nLc4, and *E. coli* P pili- Gb_3_ and Gb_4_) and (b) recombinant receptor mimics against STEC [49].

There are a variety of mechanisms used by bacterial species to exclude or reduce the growth of another species such as creation of a hostile environment, blocking available receptor sites, production and secretion of antimicrobial products and specific metabolites, and competitive depletion of essential nutrients [50]. Lactobacilli and bifidobacteria have been shown to inhibit a broad range of pathogens including *E. coli*, *Salmonella*, *Helicobacter pylori*, *Listeria monocytogenes*, and *Rotavirus* [21,51,52,53,54,55,56,57]. It has been shown that some lactobacilli and bifidobacteria compete for binding to host cell receptors because they share the same carbohydrate-binding specificities with some enteropathogens [58,59,60]. *Lactobacillus rhamnosus* has been shown to inhibit the internalization of enterohemorrhagic *E. coli* (EHEC) [61]. Generally, the ability of probiotic strains to inhibit pathogen attachment relies on steric hindrance of enterocyte pathogen receptors [62].

In Figure 4A,B, westerns blots indicate the presence of full-length BoNT/A in the presence of all strains including the probiotics. This suggests that degradation of BoNT/A is not the mechanism used by the probiotics strains to interfere with BoNT/A entry. Densitometry analysis indicates that BoNT/A remains mostly soluble in the supernatant rather than sedimenting with the bacterial/yeast insoluble pellet (Figure 4A). Thus, the non-specific binding of probiotic bacteria to cell wall constituents therefore blocking the accessibility of BoNT/A receptors could be a likely mechanism that needs further investigation.

Our results suggest that the BoNT/A internalization could be blocked by the probiotics strains of SB and some lactobacilli species. For the first time, we show a potential beneficial role that some probiotics may have in blocking the development and/or limiting the effects of human botulism. These results may lead to the development of new therapeutics for food-borne but especially relevant in regards to infant botulism. Development of a probiotic “cocktail” to initially seed the undeveloped infant gut to establish an environment of beneficial gut microbiota may protect against both *Clostridia* colonization and/or toxin absorption.

## 4. Conclusions

We show that probiotics may be beneficial in preventing the binding and internalization of botulinum neurotoxin serotype A to mammalian cells. The data suggests that the mechanism involved in this process is competitive inhibition between the probiotic strains and BoNT/A for host cell membrane receptors rather than degradation of BoNT/A or non-specific binding of toxin to the probiotics themselves.

## 5. Materials and Methods

### 5.1. Materials

Dulbecco’s modified Eagle’s medium (DMEM) (containing 4.5 g/L d-glucose and GlutaMAX), penicillin and streptomycin (100×), fetal bovine serum (FBS), TrypLE Select, Hanks’ balanced salt solution (HBSS), and phosphate buffer solution (PBS) (10×) were purchased from Life Technologies (Carlsbad, CA, USA). The human colon carcinoma cell lines (Caco-2 cells, ATCC, Manassas, VA, USA) were grown in DMEM. Cells after 50–70 passages were used in the uptake study. *L. rhamnosus* LGG (ATCC 53103), *L. reuteri* (ATCC 23272), *L*. *acidophilus* (ATCC 4356), and *Saccharomyces boulardii* (ATCC MYA-796) were obtained from the American Type Culture Collection (ATCC, Manassas, VA, USA). All other chemicals and reagents used were obtained from Sigma-Aldrich (St. Louis, MO, USA). *Escherichia coli* K12 MG1655 (EC) was obtained from Dr. Lisa Gorski at the Western Regional Research Center.

### 5.2. Growth of Yeast and Bacterial Cultures

*Escherichia coli* K12 MG1655 (EC)*, L. rhamnosus* (LGG), *L. reuteri* (Lr), *L. acidophilus* (LA), and *Saccharomyces cerevisiae* var *boulardii* (SB) were grown overnight in growth broth (LB, YPD, and MRS), washed two times with phosphate buffered saline (PBS) and resuspended in Hank’s balanced salt solution (HBSS) at either 10^4^ or 10^8^ CFU/mL. The initial concentration was determined by spectrophotometry at 600 nm and the numbers of bacteria were verified by pour-plate assay using (LB, YPD, and MRS) agar and standard serial dilution techniques.

### 5.3. Caco-2 Culture and Pre-Treatment with Probiotic Cultures

Human colonic carcinoma Caco-2 cells were grown on acid-washed 25 mm glass coverslips incubated in DMEM containing 10% FBS, 1× nonessential amino acid (NAA), and 1X penicillin and streptomycin at 37 °C in a 90% humidity and 5% CO_2_ incubator (Sanyo, Osaka, Japan). Caco-2 cells were seeded at a density of 1.2 × 10^5^ cells/well. The media was changed every two days. After five days, the cell monolayers were observed by optical microscopy (Leica Microsystems, Buffalo Grove, IL, USA) to ensure that the cells reached about 90% confluence. On the day of experiment, Caco-2 cell monolayers were washed with 1X HBSS two times and pretreated either with 10^4^ or 10^8^ EC, LGG, Lr, LA, or SC prepared in HBSS for 30 min. Non-adherent bacteria were removed from the culture with three washes of HBSS. After pretreatment, glass coverslips containing bacteria-bound Caco-2 monolayers were treated with BoNT/A complex, 50 ng·mL^−1^ (56 pM) in HBSS for 4 h. After incubation, the coverslips were washed three times with 1X PBS and fixed in 4% paraformaldehyde (PFA, Affymetrix, Santa Clara, CA, USA) for 10 min and rinsed with 1X PBS before immunofluorescence staining.

### 5.4. Immunofluorescence Staining

Fixed glass coverslips containing Caco-2 cells were rinsed twice with PBS and permeabilized with 1% triton X-100 in PBS for 30 min. Cells were incubated with blocking solution (2% goat serum, 0.2% Triton X-100, and 0.1% Bovine Serum Albumin) for 1 h, and then incubated with 1:250 blocking buffer diluted solutions of a polyclonal rabbit antibody against BoNT/A (2 mg·mL^−1^ of stock) and Rhodamine-Phalloidin (actin stain, Molecular Probes; Life Technologies). After washing three times, cells were incubated with Alexa Fluor 488 to rabbit IgG (1:500 dilution; Life Technologies) and mounted onto glass slides using the hard-set DAPI mounting medium (Vector Laboratories). Fluorescence signals from Z stacks representing the top to the bottom of optical fields were visualized with either the Leica Microsystems confocal microscope (Leica TCS SP5) or with Zeiss Axio Observer.Z1 microscope with Apotome.2 with the appropriate filters set at 40× magnification. Negative controls were prepared by omitting the primary antibodies.

### 5.5. Western Blotting

Western blotting was performed to evaluate the probiotic mechanism of action with the BoNT/A neurotoxin. *E. coli, L. rhamnosus*, *L. reuteri*, *L. acidophilus*, and *Saccharomyces boulardii* were grown overnight in growth media (LB, YPD, and MRS). One mL of bacteria was washed three times with 1X HBSS and resuspended in 1 mL of HBSS. Aliquots of 50 μL bacteria mixture were taken from each strain and put in 1.5 mL Eppendorf Lo-bind tubes. Each tube containing bacteria was treated with 2 μL of 1 mg/mL BoNT/A complex for 4 h at 37 °C in a 90% humidity and 5% CO_2_ incubator. After incubation, the tubes were centrifuged at 2000 × *g* for 5 min and the supernatant and pellet were separated. Protein samples were TCA precipitated and separated by sodium dodecyl sulfate (SDS)-polyacrylamide gel electrophoresis (PAGE) with NuPAGE 10% Bis-Tris gels (Invitrogen) followed by Western blotting. The resolved proteins were transferred to a PVDF membrane (Immobilon). The membrane was blocked in 5% milk-Tris-buffered saline-0.05% Tween 20 buffer then probed with polyclonal rabbit anti-BoNT/A antibody (2 mg·mL^−1^ of stock) diluted to 1:2000 with blocking solution followed by secondary antibody (Horseradish peroxidase (HRP)-conjugated; 1:2000). The blot was incubated in Pierce ECL Western Blotting Substrate solution (Thermo Scientific). Protein bands from peroxidase activities to chemiluminescent substrates were developed and detected using the FluorChem SP AlphaImager (Alpha Innotech, San Leandro, CA, USA). Molecular weight standards were purchased from Invitrogen. Densitometry was performed using the FluorChem analysis software. Percent of BoNT/A signal from the soluble and pellet was quantified from four independent experiments and plotted using GraphPad Prism 6.

### 5.6. Statistics

For the cell culture studies, *n* = number of independent experiments, each independent experiment contained triplicate culture wells with one coverslip per each study condition. BoNT/A signal, representing the total fluorescence intensity in the cells calculated from area multiplied by mean intensity, was quantified from at least 30 optical fields (Z stacks) taken from four independent experiments. For quantification, the mean fluorescence was measured in at least three randomly selected non-overlapping 40× fields with each containing approximately 100–150 Caco-2 cells. All data were expressed as mean ± standard error of the mean (SEM) and assessed using two-way ANOVA followed by the Tukey-Kramer test where multiple groups are compared with *p* values < 0.05 are taken to indicate significant differences between groups. Western data was assembled from four independent experiments and statistical significance was determined by two-tailed unpaired Student’s *t*-test.

## Abbreviations

The following abbreviations are used in this manuscript:

BoNTs	Botulinum neurotoxins
NAPs	neurotoxin-associated proteins
CFU	colony forming unit
PBS	phosphate buffered saline
